# Genome-wide detection of *Wolbachia* in natural *Aedes aegypti* populations using ddRAD-Seq

**DOI:** 10.3389/fcimb.2023.1252656

**Published:** 2023-12-14

**Authors:** Atikah Fitria Muharromah, Jerica Isabel L. Reyes, Ngure Kagia, Kozo Watanabe

**Affiliations:** ^1^ Molecular Ecology and Health Laboratory, Center for Marine Environmental Studies (CMES), Ehime University, Matsuyama, Japan; ^2^ Entomology Laboratory, Department of Tropical Biology, Faculty of Biology, Universitas Gadjah Mada, Yogyakarta, Indonesia

**Keywords:** *Wolbachia*, *Aedes aegypti*, ddRAD-seq, Philippines, genome-wide

## Abstract

**Background:**

*Wolbachia*, an endosymbiotic bacterium, is globally used to control arboviruses because of its ability to block arboviral replication and manipulate the reproduction of *Wolbachia* host, *Aedes aegypti*. Polymerase chain reaction (PCR)-based *Wolbachia* detection has been recently reported from natural *Ae. aegypti* populations. However, due to the technical limitations of PCR, such as primer incompatibility, PCR-based assays are not sufficiently reliable or accurate. In this study, we examined double digestion restriction site-associated DNA sequencing (ddRAD-Seq) efficiency and limitations in *Wolbachia* detection and quantification in field-collected *Ae. aegypti* natural populations in Metro Manila, the Philippines, compared with PCR-based assays.

**Methods:**

A total of 217 individuals *Ae. aegypti* were collected from Metropolitan Manila, Philippines. We separated it into 14 populations consisting of 7 female and male populations. We constructed a library for pool ddRAD-Seq per population and also screened for *Wolbachia* by PCR assays using *wsp* and *16S* rRNA. *Wolbachia* density per population were measured using *RPS17* as the housekeeping gene.

**Results:**

From 146,239,637 sequence reads obtained, 26,299 and 43,778 reads were mapped across the entire *Wolbachia* genome (with the *w*AlbA and *w*AlbB strains, respectively), suggesting that ddRAD-Seq complements PCR assays and supports more reliable *Wolbachia* detection from a genome-wide perspective. The number of reads mapped to the *Wolbachia* genome per population positively correlated with the number of *Wolbachia*-infected individuals per population based on PCR assays and the relative density of *Wolbachia* in the *Ae. aegypti* populations based on qPCR, suggesting ddRAD-Seq-based semi-quantification of *Wolbachia* by ddRAD-Seq. Male *Ae. aegypti* exhibited more reads mapped to the *Wolbachia* genome than females, suggesting higher *Wolbachia* prevalence rates in their case. We detected 150 single nucleotide polymorphism loci across the *Wolbachia* genome, allowing for more accurate the detection of four strains: *w*Pip*, w*Ri*, TRS of Brugia malayi*, and *w*Mel.

**Conclusions:**

Taken together, our results demonstrate the feasibility of ddRAD-Seq-based *Wolbachia* detection from field-collected *Ae. aegypti* mosquitoes.

## 1 Introduction

Dengue, Zika, and Chikungunya represent major public health concerns worldwide (Silva et al., 2020). These arboviral diseases are transmitted by the vector mosquito *Aedes aegypti*. A novel approach for combating these mosquito-borne diseases using *Wolbachia* bacteria has been established in various countries (Hoffmann et al., 2011; Nguyen et al., 2015; Schmidt et al., 2017; Nazni et al., 2019; Zheng et al., 2019; Crawford et al., 2020; Beebe et al., 2021; Pinto et al., 2021; Utarini et al., 2021; Ahmad et al., 2021). Exploiting the cytoplasmic incompatibility of *Wolbachia* on host reproduction could suppress mosquito populations by resulting in an unviable embryo, thereby replacing natural mosquito populations with *Wolbachia*-infected ones. For instance, dengue incidences in *Wolbachia*-treated areas in Australia and Indonesia were reduced by 96% (Ryan et al., 2020) and 77% (Utarini et al., 2021), respectively.

*Wolbachia* inhibits arboviral replication in host mosquitoes. The *w*Mel *Wolbachia* strain reduces CHIKV (Aliota et al., 2016b), ZIKV (Aliota et al., 2016a), and DENV (Walker et al., 2011) transmission from *Ae. aegypti* to other hosts, including humans. Another *Wolbachia* strain, *w*AlbA, blocks ZIKV (Chouin-Carneiro et al., 2019) and the *w*AlbB strain might inhibit dengue and Zika virus transmission in *Ae. aegypti* (Hugo et al., 2022). For an effective *Wolbachia*-based arbovirus control, important information such as *Wolbachia* infection prevalence and that related to natural mosquito population strains should be available, as *Wolbachia* strain co-infection in *Ae. aegypti* could potentially induce inter-strain competition in the host mosquito. For example, triple-strain infection (*w*Mel, *w*AlbA, and *w*AlbB) in *Ae. albopictus* and *Ae. aegypti* inhibited cytoplasmic incompatibility expression and showed low maternal transmission fidelity (Ant and Sinkins, 2018; Liang et al., 2020). Prior knowledge of *Wolbachia* strains in native mosquito populations in the deployment area could help identify the most suitable *Wolbachia* strain to use.

Natural *Wolbachia* infection in *Ae. aegypti* remains controversial. For instance, using PCR assays, Gloria-Soria et al. (2018) did not detect *Wolbachia* in any *Ae. aegypti* collected from 27 countries. Other studies also confirmed the lack of *Wolbachia* detection in *Ae. aegypti* from Cape Verde islands (da Moura et al., 2023), Singapore (Ding et al., 2020) and California (Torres et al., 2020). In contrast, natural *Wolbachia* infection could be identified in *Ae. aegypti* in the USA (Coon et al., 2016; Kulkarni et al., 2019), Malaysia (Teo et al., 2017), Thailand (Thongsripong et al., 2017), India (Balaji et al., 2019), the Philippines (Carvajal et al., 2019), Panama (Bennett et al., 2019), and China (Zhang et al., 2022). A possible explanation for these results is the different susceptibility to *Wolbachia* between host individuals, potentially influenced by host genotype and environmental conditions (Mouton et al., 2007). False positive detections due to *Wolbachia* contamination from other mosquito host species during the larval stage could also be suspected. False negative detections could be due to PCR primer incompatibility or low bacterial concentration of *Wolbachia* in the mosquito. For example, a previous study described host age- and sex-related *Wolbachia* density variances in mosquito bodies (Tortosa et al., 2010). Further data would be required to validate natural *Wolbachia* infection in *Ae. aegypti*.

Polymerase chain reaction (PCR) is frequently used to diagnose *Wolbachia* infection in insects (de Oliveira et al., 2015). Independent PCR tests for multiple *Wolbachia* genes would be encouraged to reduce the possibility of false negative results. In addition, the authors of previous studies involving PCR assays did not use locally designed primers specific to the tested local *Wolbachia* populations, potentially resulting in false negative detection due to primer incompatibility if large genetic variations were present in the target genes among local populations. To design such local primers for multiple genes, prior genomic information on the local *Wolbachia* population would be highly desirable, but such prior information is usually not available.

High-throughput sequencing technologies, such as double digestion restriction site-associated DNA sequencing (ddRAD-Seq), could serve as a powerful alternative to address these limitations. DdRAD-Seq uses two different restriction enzymes (RE) to segment the whole genome of organisms into short fragments (Peterson et al., 2012), and sequences numerous randomly selected DNA fragments in parallel. When using DNA extracted from *Ae. aegypti* individuals, the genome of the microorganisms present in the mosquito would also be sequenced. The ddRAD-Seq provides genome-wide information without requiring prior knowledge of the local target populations, potentially reducing PCR primer incompatibility-related false negative detection. So far, few studies have used ddRAD-Seq to detect *Wolbachia*. Lee et al. (2020) used ddRAD-Seq to observe the coevolution of *Wolbachia* and its host, *Anoplolepis gracilipes*. Yang et al. (2022) characterized *w*AlbA and *w*AlbB *Wolbachia* strain infection in *Ae. albopictus* using ddRAD-Seq. However, to the best of our knowledge, no study has applied ddRAD-Seq to detect *Wolbachia* in *Ae. aegypti*.

Sequencing the microorganism endosymbiont DNA extracted from *Ae. aegypti* might allow the detection of *Wolbachia* DNA sequences. In this study, we examined the feasibility of using ddRAD-Seq for detecting and quantifying *Wolbachia* from field-collected female and male *Ae. aegypti* populations in Metropolitan Manila, the Philippines. We assessed the accuracy of *Wolbachia* detection using ddRAD-Seq compared to the results of PCR assays (both conventional and quantitative PCR) and explored the advantages and limitations of these methods in *Wolbachia* detection and quantification. We also estimated the genetic diversity of *Wolbachia* in field-collected *Ae. aegypti* samples using ddRAD-Seq-derived reads.

## 2 Materials and methods

### 2.1 Mosquito sampling

**Figure 1:**
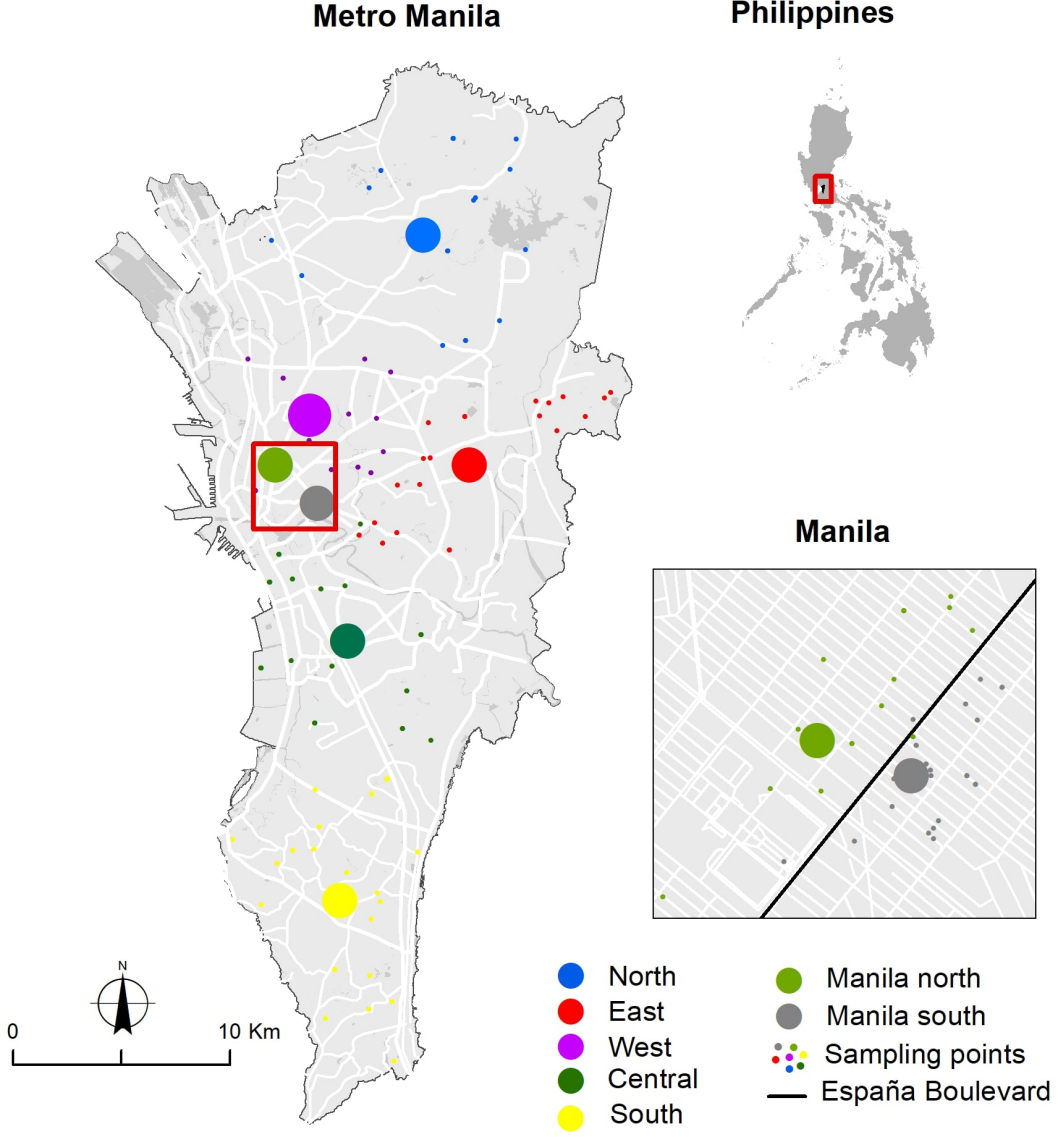
Sampling site locations of *Ae. aegypti* in Manila City, Metropolitan Manila, Philippines. Big circles in red, yellow, dark green, light green, gray, dark blue, and purple indicate the geographical midpoints of *Ae. aegypti* populations per location; small circles near each big circle indicate the households in the sampling locations. F and M indicate the total number of female and male individuals per population, respectively (Muharromah et al., 2023).

*Ae. aegypti* mosquitoes were collected from Metropolitan Manila, the Philippines. A total of 217 *Ae. aegypti* individuals (93 males and 124 females) that had been previously used by Carvajal et al. (2020) and Regilme et al. (2021) were used in this study. We assessed male and female populations from seven different regions in Metropolitan Manila (**Figure 1**). Adult mosquitoes were collected using a UV light trap (Mosquito Trap, Jocanima Corporation, Las Pinas City, Philippines) from May 2014 to January 2015 (Carvajal et al., 2020) and from September to October 2017 (Regilme et al., 2021). Mosquito identification was conducted using pictorial keys from Rueda (2004) and the molecular method using species-specific microsatellite markers undertaken by previous studies (Carvajal et al., 2020; Regilme et al., 2021). The current study used the same DNA samples as the two previous studies (Carvajal et al., 2020; Regilme et al., 2021) to detect and quantify *Wolbachia*.

### 2.2 DNA preparation, ddRAD-sequencing, and data processing

The ddRAD-Sequencing data were obtained from (Muharromah et al., 2023). For the library construction, firstly, the DNA sequences of each *Ae. aegypti* mosquito was determined using a Quantus Fluorometer (Promega, USA). Individual DNA samples of all 14 populations were pooled in equimolar DNA amounts (Pool-Seq) (Schlötterer et al., 2014) based on the sex (female and male) and location (Central, East, West, North, South, Manila North and Manila South). Prior to the library preparation, we optimized in selecting the restriction enzymes for ddRAD-Seq. The restriction enzymes for ddRAD-Seq were selected using two approaches: *in silico* and empirical approach. We compared seven restriction enzyme combinations (*DraI-NlaIII, MluCI-NlaIII* (Rašić et al., 2014), *DraI-MluCI, SbfI HF-MspI* (Sherpa et al., 2018), *EcoRI-NlaIII* (Rašić et al., 2014) *SbfI HF-HaeIII* (Gamboa and Watanabe, 2019), and *SspI-NlaIII*) to produce desired sequenceable DNA fragments of c.a. 100–500 bp, which following the addition of adapters and sequencing primers will result in an acceptable library size for sequencing (c.a. 200–700 bp). Double digestion with *in silico* analysis allows the prediction of the number of sequenceable DNA fragments using restriction-site information from the enzymes and reference genome of *Ae. aegypti* via the *DDsilico* program (Rašić et al., 2014). Empirical digestion analysis is an experimental method for observing DNA fragment distribution using the actual DNA of *Ae. aegypti* and restriction enzymes visualized with a High-Sensitivity DNA Assay 2100 Bioanalyzer (Agilent, USA). We selected *MluCI* and *NlaIII* (New England Biolabs, Beverly MA, USA) as the optimal combination because it generated the highest number of potential ddRAD loci using the *in silico* and empirical approaches (**Supplementary Figures 1**, **2**). The ddRAD-Sequencing library preparation was performed using the Restriction Enzymes (REs) *NlaIII* and *MluCI* (New England Biolabs, USA) (Rašić et al., 2014) to digest the *Ae. aegypti* DNA for 3 h at 37°C. Next, the REs were inactivated at 65°C for 20 min and purified using a QiaQuick PCR Purification Kit (Qiagen, Hilden, Germany). The digested DNA was ligated to Illumina P1 and P2 adapters using a T4 Ligation mix containing 0.5 µl of 4 nM/µl P1 Adapter, 0.5 µl of 6 nM/µl P2 Adapter, T4 DNA ligase (Takara Bio, Japan), T4 ligase buffer and H_2_O at 16°C for 16 h with the total volume 15 µl, after which the ligase was inactivated at 65°C for 20 min. The adapter-ligated DNA was amplified in a 10-µl PCR reaction mix containing 5 µl of Phusion High Fidelity Master Mix (New England Biolabs, USA), 2 µl of P1 primer (5’-AATGATACGGCGACCACCGAGATCTACACTCTTTCCCTACACGACG-3’), and 2 µl of P2 primer (5’-CAAGCAGAAGACGGCATACGAGATCGTGATGTGACTGGAGTTCAGACGTGTGC- 3’) with the PCR cycling conditions as follows: 98°C for 30 s; 12 cycles of 98°C for 10 s, 60°C for 30 s, and 72°C for 90 s; and elongation at 72°C for 5 min. The final library was formed by pooling seven PCR replicates and purified using a Qiaquick PCR Purification Kit (Qiagen, Hilden, Germany). The library was checked for quality and quantity using Bioanalyzer (Agilent Technologies, USA) and KAPA Quantification kits (Roche, USA). After that, the library was sequenced using a HiSeq X Ten Illumina sequencer (paired-end, 2 × 150 bp) at the Beijing Genomics Institute, China.

The raw sequence data were verified for quality using FASTQC v0.11.8 (Andrews, 2010). The reads were trimmed and filtered to remove the adapters and the barcodes using Trimmomatics 0.39 (Bolger et al., 2014), retaining 100 bp of read length. The reads were mapped to the *Wolbachia* reference genome (Sinha et al., 2019; Martinez et al., 2022) using the *bwa mem* algorithm in BWA (Li and Durbin, 2009), generating a SAM format file per population. The ambiguously mapped reads from the mapping were filtered with a minimum MAPQ score of 20. This MAPQ score indicated the possibility that less than 1 out of 100 mappings were incorrect. The SAM files were converted to BAM files using SAMTOOLS 1.9 (Danecek et al., 2021) to have a memory-efficient file form. The reads mapped to the *Wolbachia* genome were extracted using the *samtools* view command in SAMTOOLS 1.9. The extracted reads were sorted toward the reference coordinates using SAMTOOLS 1.9. Calling single nucleotide polymorphisms (SNPs) was conducted using bcftools (Li, 2011; Danecek et al., 2021). First, all of population files were merged using bcftools mpileup command. We converted the bcf file to vcf file form using bcftools filter command. The SNPs were filtered using bcftools for minimum quality 20 and minimum read depth of 10. After that, the nucleotide diversity were calculated using vcftools (Danecek et al., 2011) over 10 kb windows of the genome (nucleotide diversity value was estimated for every 10,000 bases across the genome). To identify the species and strain obtained in each mosquito population, the sorted BAM file per population was converted to FASTA file format, then identified using MMSeqs2 version 13.45111 (Steinegger and Söding, 2017) using the UniProtKB/SwissProt database (Bairoch and Apweiler, 2000) released in 27 April 2022 by comparing the amino acids from the query sequence with those from the database and high sensitivity value (-s 9) to improve accuracy. To visualize the mapped read in the *Wolbachia* genome, we used Proksee (Stothard et al., 2019). The gene annotation was performed using Prokka 1.14.6 (Seemann, 2014) provided in Proksee.

In identifying other bacteria from the samples, we classified the raw reads using Kaiju (Menzel et al., 2016) with the NCBI non-redundant (NR) database and minimum occurrence percentage ≥0.0001%. We calculated the number of *Wolbachia* contigs by *de novo* assembly from the filtered data using metaSPAdes (Nurk et al., 2017) and then we classified it using Kaiju with NCBI non-redundant database.

### 2.3 PCR for *Wolbachia* detection and quantification

The PCR-based *Wolbachia* detection/non-detection data (targeting the *16S* rRNA and *wsp* genes of each individual in the ddRAD analysis) were obtained from Reyes et al. (2022) for samples from 10 populations in Metropolitan Manila. For this, a *16S* rRNA and *wsp* gene marker has been used (**Table 1**). In addition, the PCR data results of four populations in Manila City were obtained from Regilme et al. (2022) using the same target gene markers (**Table 1**). In addition, we also performed quantitative PCR (qPCR) assays targeting the *wsp* gene in the pooled DNA samples of each population for ddRAD using Real-Time Quantitative PCR (Bio-Rad, USA). In this pool-based qPCR, we used primers designed by Reyes et al. (2022) for detection of the *wsp* gene from each mosquito individual comprising the 14 populations of Metropolitan Manila. Reyes et al. (2022) designed the primers for 118 *wsp* sequences extracted from *Ae. aegypti* samples and sequenced by Carvajal et al. (2019) (GenBank popset 1712729902). Next, Multiple Sequence Comparison by Log-Expectation was used for multiple sequence alignment and Codon Code Aligner version 1.2.4 (available at https://www.codoncode.com/aligner/) to display the outcomes. The consensus sequence of the alignment was then used to create *wsp* primers for the *Ae. aegypti* samples using Primer-BLAST. Five primer pairs were produced using Primer-BLAST and they were confirmed using a known positive sample of *Cx. quinquefasciatus*. Two of five primer pairs (wspAAML 01 and wspAAML 05) were chosen from the group for further optimization as they yielded the proper band size of the target markers in the sample without any nonspecific binding. Then, Reyes et al. (2022) established the optimal annealing temperature and primer concentration for both pairings to select the best wspAAML primer pair for further investigation. After careful consideration, *wsp* 05 was chosen since its PCR efficiency was within the typical MIQE criterion of ≥90%. This approach enabled the design of primers capable of detecting the variable sequences of *wsp* genes present in the local populations of *Wolbachia* in this region.

**Table 1:** PCR primers used for *Wolbachia* detection.

Name	Gene	Oligonucleotide sequence (5’-3’)	Probe	Reference
16SF	*16S* rRNA	5′-AGTGAAGAAGGCCTTTGGG-3′	5′TET-CTGTGAGTACCGTCATTATCTTCCTCACT-BHQ13′	Fraser et al. (2020)
16SR	*16S* rRNA	5′-CACGGAGTTAGCCAGGACTTC-3′		
wspAAML F	*wsp*	5′-AGCATCTTTTATGGCTGGTGG-3′	5′FAM-ACGACGTTGGTGGTGCAACATTTGC-TAMRA3′	Reyes et al. (2022)
wspAAML R	*wsp*	5′- AATGCTGCCACACTGTTTGC-3′		
WolbF	*16S* rRNA	5′-GAAGATAATGACGGTACTCAC-3′		Zhou et al. (1998)
Wspecr	*16S* rRNA	5′-AGCTTC GAGTGAAACCAATTC-3′		
*wsp* 81F	*wsp*	5′-TGGTCCAATAAGTGATGAAGAAAC-3′		Simoes et al. (2011)
*wsp* 691R	*wsp*	5′-AAAAATTAAACGCTACTCCA-3′		
17SF	*RPS17*	5′-TCCGTGGTATCTCCATCAAGCT-3′	5′HEX-CAGGAGGAGGAACGTGAGCGCAG-BHQ13′	Frentiu et al., 2014
17SR	*RPS17*	5′-CACTTCCGGCACGTAGTTGTC-3′		

The relative density of *Wolbachia* was calculated using the *Ae. aegypti* ribosomal S17 (*RPS17*) gene as a housekeeping/reference gene (**Table 1**). The relative density of *Wolbachia* was assessed using the delta CT calculation method (where CT refers to the qPCR threshold cycle) as follows: 2 ^CT^(using the *RPS17* reference gene)/ 2 ^CT^(target *16S* rRNA or *wsp* genes) (Fraser et al., 2017). For the *16S* rRNA gene amplification, we used 5 µl of 1X iTaq mix (Bio-Rad) with 0.2 µl of 0.2 µM *16S* rRNA primers. The *16S rRNA* reaction was performed in a volume of 10 µl, using 0.15µl of the probe at a concentration of 0.15 µM and completing the reaction with 3.45 µl of H_2_O. Concerning the *wsp* amplification, we also used 5 µl of iTaq mix (Bio-Rad) at 1x concentration with 0.5 μM of *wsp* forward and reverse primers in a volume of 0.5 μl per primer, 0.3 µM of *wsp* probe at a volume of 0.3 µl, and 2.7 µl of H_2_O to obtain a total reaction volume of 10 µl. The *RPS17* gene was amplified using 5µl of iTaq mix (Bio-Rad) at 1x concentration, 0.3 µl of 0.3 µM forward and reverse *RPS17* primers, 0.2 µl of *RPS17* gene probe at 0.2 µM, and 3.2 µl of H_2_O were added to complete the reaction volume to a total of 10 µl. The PCR cycling conditions for the *RPS 17* and *16S* rRNA genes were as follows: 95°C for 30 s; 95°C for 5 s; 60°C for 10 s with 40 cycles. The PCR cycling conditions for the *wsp* gene were as follows: 95°C for 2 min; 95°C for 30 s; 58.8°C for 30 s with 40 cycles.

### 2.4 Data analysis

The total number of PCR-detected *Wolbachia*-positive individuals for the *16S* rRNA and *wsp* genes per population was standardized by dividing it by the total number of analyzed individuals per population. The total number of reads mapped to the *Wolbachia* genome per population was also standardized by dividing it by the total number of reads remaining after quality filtering using Trimmomatics per population. The correlation between the percentage of ddRAD-Seq reads mapped to the *Wolbachia* genome per population and the percentage of *Wolbachia*-positive individuals detected by PCR per population was examined using Spearman’s correlation test in RStudio version 1.4.1106. Similarly, we tested the correlation between the percentage of reads mapped to the *Wolbachia* genome per population, measured the relative *Wolbachia* density per population by qPCR assays, and analyzed the total number of individuals by ddRAD per population using Spearman’s correlation test.

## 3 Results

### 3.1 *Wolbachia* detection using pooled ddRAD-sequencing

ddRAD-Seq produced a total of 377,047,648 raw reads with an average of 26,931,975 reads per population. After quality filtering and trimming, we obtained a total of 146,239,637 reads with a minimum length of 100 bp. The ddRAD-Seq data showed varying numbers of reads mapped to the *Wolbachia w*AlbA and *w*AlbB genomes among the 14 *Ae. aegypti* populations in Metropolitan Manila (**Table 2**). Ten populations (Female and Male Central, East, North, South, and West) were confirmed to display *Wolbachia* genome sequences with more than 100 reads mapped to the *Wolbachia* genome. However, four populations from Manila City were detected to exhibit a few reads mapped to *Wolbachia* (< 100 reads). The highest read number mapped to the *Wolbachia* genome was found in the female South population, followed by the male West and male South populations. We observed a higher total number of reads mapped to the *w*AlbB genome compared to that to the *w*AlbA genome. In the following analysis, we only used the reads mapped to the *w*AlbB genome.

**Table 2:** The number of reads obtained by ddRAD-Seq analysis in 14 populations of *Ae. aegypti* and the number of the reads that mapped to the *w*AlbA or *w*AlbB reference genomes and all bacteria classified using Kaiju.

No	Population	N	Reads	*w*AlbA	*w*AlbB	All bacteria classified reads using Kaiju
**1**	F_Central	24	13,278,566	502	553	247,145 (0.74%)
**2**	F_East	19	10,486,484	86	88	33,201 (0.11%)
**3**	F_North	12	8,938,915	1,016	1,643	68,807 (0.33%)
**4**	F_South	28	8,938,153	2,480	14,459	34,386 (0.14%)
**5**	F_West	18	10,250,794	192	199	68,524 (0.25%)
**6**	F_North_Manila	12	7,469,088	17	17	2,224 (0.012%)
**7**	F_South Manila	16	9,624,898	75	77	5,658 (0.02%)
**8**	M_Central	20	10,250,794	941	1,925	117,544 (0.59%)
**9**	M_East	12	8,056,812	374	473	57,652 (0.26%)
**10**	M_North	7	8,572,293	215	211	68,895 (0.38%)
**11**	M_South	20	14,003,836	8,588	11,509	72,053 (0.24%)
**12**	M_West	12	11,627,817	11,747	12,547	41,987 (0.14%)
**13**	M_North_Manila	15	10,280,531	13	13	3,485 (0.013%)
**14**	M_South_Manila	9	14,460,656	53	64	12,296 (0.03%)
**Total**		217	146,239,637	26,299	43,778	833,857

N = total number of analyzed individuals, Reads = number of reads obtained after trimming and filtering, *w*AlbA and *w*AlbB = total number of reads mapped to the wAlbA and wAlbB reference genomes, respectively.

### 3.2 Detection accuracy of ddRAD-sequencing compared with the PCR assays

A total of 217 samples used in this study were tested using PCR assays both on individual and pooled data. **Table 2** shows the results of *Wolbachia* detection and relative density using the PCR assays. The PCR assay results indicated that 85 and 49 individuals from the 217 samples could be positively detected with *Wolbachia* using the *wsp* and *16S* rRNA genes, respectively. The relative density of *Wolbachia* per individual was in the range of 0.0002–147.03 (for *16S* rRNA) and 0.0002–64.44 (for *wsp*). The qPCR results on the pooled DNA samples showed positive results for seven populations (Female Central, North, and South; Male Central, East, South, and West) with the relative density of *Wolbachia* in the range of 0.010–1.51 (**Table 3**).

**Table 3:** Individual-based detection of *Wolbachia* in *Ae. aegypti* using PCR and relative density *Wolbachia* using individual-based and pool-based estimation.

Population	N	*wsp*	*16S* rRNA	*wsp, 16S* rRNA	X̄ Relative Density (*wsp*)- Ind	X̄ Relative Density (*16S* rRNA)- Ind	Relative Density (*wsp*)-Pool
**F_Central**	24	15	8	7	0.158	0.015	0.669
**F_East**	19	6	2	1	0.026	0.002	0
**F_North**	12	3	2	2	0.194	0.160	0.146
**F_South**	28	13	13	11	0.019	2.184	0.028
**F_West**	18	7	4	3	0.003	0.001	0
**F_North_Manila**	12	0	0	0	0	0	0
**F_South Manila**	16	0	0	0	0	0	0
**M_Central**	20	14	6	5	0.039	3.702	0.170
**M_East**	12	5	2	2	0.036	0.003	0.010
**M_North**	7	1	1	1	0.002	0.015	0
**M_South**	20	11	9	9	1.470	3.046	1.505
**M_West**	12	8	2	2	8.145	73.776	0.551
**M_North_Manila**	15	0	0	0	0	0	0
**M_South_Manila**	9	0	0	0	0	0	0
* Total	217	83	49	43			

N = total number of individuals, x̄ = mean value.

**Figure 2:**
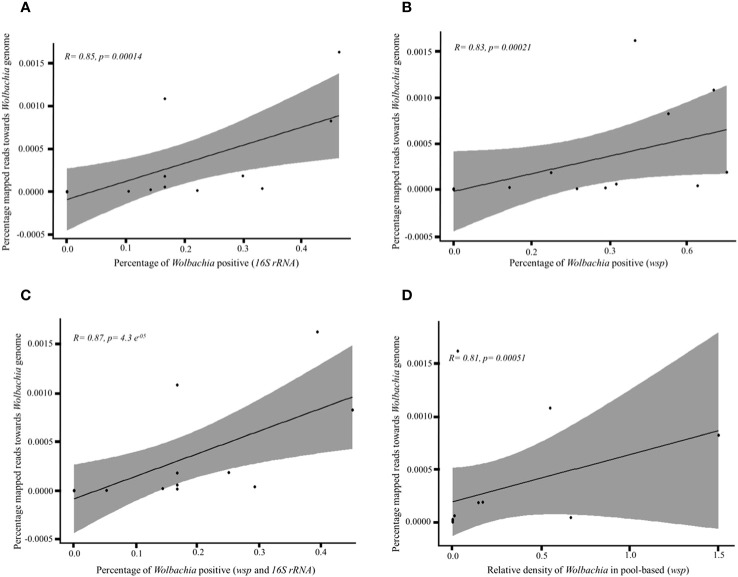
Correlation plots between the percentage of mapped reads to the *Wolbachia* genome and that of *Wolbachia*-positive mosquitoes with *16S* rRNA gene **(A)**, *wsp* gene **(B)**, both *16S* rRNA and *wsp* genes **(C)**, and the relative *Wolbachia* density in the pooled data **(D)**. Percentage of reads mapped in the *Wolbachia* genome = total number of reads mapped in the *Wolbachia* genome divided by the total number of reads after trimming and filtering, percentage of *Wolbachia*-positive mosquitoes = total number of *Wolbachia*-positive individuals divided by the total number of individuals per population.

The percentage of *Wolbachia*-positive individuals detected by PCR per population showed a positive correlation with the percentage of ddRAD-Seq reads mapped to the *Wolbachia* genome per population both for the *16S* rRNA (**Figure 2A**, *p* = 0.0001) and *wsp* (**Figure 2B**, *p* = 0.0002) gene. Furthermore, the percentage of *Wolbachia*-positive individuals detected using both the *16S* rRNA and *wsp* genes also positively correlated with the percentage of reads mapped to the *Wolbachia* genome (**Figure 2C**, *p* < 0.0001). The relative *Wolbachia* density per population measured by qPCR also showed a positive correlation with the percentage of ddRAD-Seq reads mapped to the *Wolbachia* genome both for the 16*S* rRNA (**Supplementary Figure 1A**, *p* < 0.0001) and *wsp* (**Supplementary Figure 1B**, *p* = 0.0004) gene. For the pooled data, the percentage of mapped reads toward the *Wolbachia* genome showed a positive correlation with the relative *Wolbachia* density estimated by qPCR (**Figure 2D**, *p* = 0.0005). Finally, the percentage of reads mapped to the *Wolbachia* genome per population did not correlate with the total number of individuals analyzed with ddRAD per population (*p* > 0.05) (**Supplementary Figure 1C**).

### 3.3 The genetic diversity of *Wolbachia*

**Figure 3:**
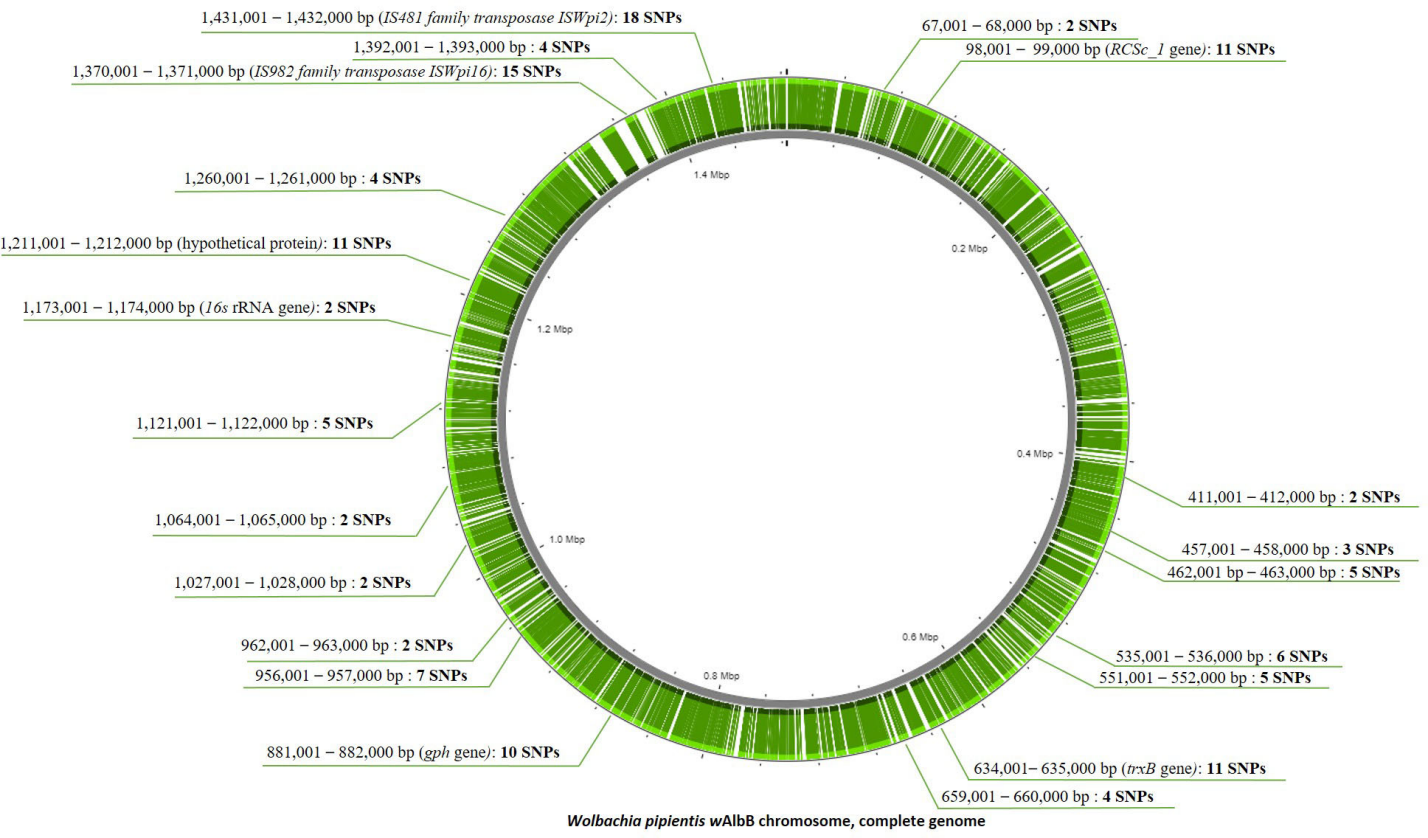
The visualization of sequence reads generated based on the ddRAD-Seq approach (green) and gap regions (white) on the *Wolbachia pipientis w*AlbB complete genome. The green arrow indicates the region of single nucleotide polymorphisms (SNPs) located across the *Wolbachia pipientis w*AlbB complete genome. **Supplementary Table 2** includes detailed information about the 150 SNPs.

A total of 150 SNPs were detected from the cumulative reads mapped to the *Wolbachia* genome from the 14 *Ae. aegypti* populations. We observed low nucleotide diversity from these SNPs (π = 0.00000651). A total of 21 regions in the 10,000 bp of sliding windows in the *Wolbachia* genome showed an SNP number in the range of 2–18. Only two SNPs were found in the conserved *16S rRNA* gene region (**Figure 3**). A high number of SNPs (SNPs > 10) could be observed in 6 of the 21 regions (**Supplementary Table 2**). These regions were further annotated and the regions of 98,001–99,000 bp (11 SNPs), 634,001–635,000 bp (11 SNPs), 881,001–882,000 bp (10 SNPs), 1,211,001–1,212,000 bp (11 SNPs), 1,370,001–1,371,000 bp (15 SNPs), and 1,431,001–1,432,000 bp (18 SNPs) were located in the *RCSc_1, trxB, gph*, *hypothetical protein, IS982 family transposase ISWpi16, IS481 family transposase ISWpi2* genes, respectively.

From the reads mapped to the *Wolbachia* genome, we identified the following four *Wolbachia* strains using MMSeqs2 (*w*Pip, *w*Ri, *TRS* of *Brugia malayi*, and *w*Mel). One of the four strains was from two *Wolbachia* species (*Wolbachia endosymbiont Culex quinquefasciatus* and *Wolbachia pipientis*), while another strain (*w*Ri) could not be identified at the species level as the DNA sequence searched from the database did not indicate the species name (**Supplementary Table 1**).

### 3.4 Diversity of bacteria identified from the ddRAD-Seq reads and number of *Wolbachia* contigs using kaiju

The bacteria diversity in the *Ae. aegypti* populations were different from each other (**Supplementary Table 3**). The percentage of all bacteria reads from the raw reads of all 14 population samples is 0.22% with the *Wolbachia* reads percentage is 0.00327%. We observed the percentage of bacteria in each *Ae. aegypti* per population is less than 1%. The higher bacteria diversity is in F Central population with the percentage 0.74% and the lowest bacteria diversity is in F North Manila (0.012%) (**Table 2**). The *Wolbachia* contigs from our data showed high number in the population of F Central, F North, F South, M Central, M South and M West (**Supplementary Table 4**).

## 4 Discussion

In this study, we tested the feasibility of using ddRAD-Seq for *Wolbachia* detection and quantification from field-collected *Ae. aegypti* populations in Metropolitan Manila, the Philippines. Overall, the number of ddRAD-Seq reads mapped to the *Wolbachia* genome in each population showed a consistent pattern with the results of PCR- and qPCR-based *Wolbachia* detections and quantifications. As expected, the ddRAD-Seq reads revealed numerous *Wolbachia* genes across the entire genome. This result suggests that ddRAD-Seq might complement conventional *Wolbachia* detection PCR assays that rely only on a few DNA markers, thereby providing stronger and more reliable support for genome-wide *Wolbachia* detection. The ddRAD-Seq approach enabled us to obtain information on a large number of genes randomly sampled from the *Wolbachia* genome without using *Wolbachia*-specific primers. Therefore, theoretically, it could be expected to reduce false negative detections due to primer incompatibility for genetically diverse populations using PCR. The *Wolbachia* detection based on the PCR assay targets only a limited number of loci (e.g., *16S* rRNA or *wsp*). ddRAD-Seq targets a large number of loci randomly selected from the *Wolbachia* genome, which increases the possibility of detection at any of the loci. In support of this theory, we found *Wolbachia* sequences from ddRAD-Seq reads in a population in Manila City, where the PCR assays did not detect *Wolbachia*, although the number of mapped reads was small (<100 reads) (**Table 2**).In addition, while PCR-based assays did not detect *Wolbachia* from the populations of Female West and Male North (**Table 3**), the ddRAD-Seq detected sequences of *Culex quinquefasciatus Pel Wolbachia endosymbiont* and unclassified *Wolbachia* from these populations (**Supplementary Table 1**), which are not *16S* rRNA and *wsp* genes in the *Wolbachia* genome.

However, the ddRAD-Seq approach also has limitations in detecting and quantifying *Wolbachia*. One is the possibility of false positive detection. The ddRAD-Seq randomly generates DNA sequence fragments of the organisms. Therefore, ddRAD-Seq reads from other bacteria evolutionarily close to *Wolbachia* could be mistakenly identified as *Wolbachia*, especially if the evolutionary rate of that sequence region is low (i.e., no/small interspecific variation). Although we tried to remove such ambiguously mapped reads after mapping the reference genome and increasing the sensitivity criteria in the identification using MMSeqs2, this possibility still cannot be completely excluded. Furthermore, mechanical errors could still occur related to the ddRAD-Seq data generated by the Illumina platform even after quality filtering, and thus the occurrence of erroneous sequences unintentionally identified as *Wolbachia* cannot be completely prevented. In addition, it is known that *Wolbachia* transfer its genes to the host genome (Kondo et al., 2002; Sieber et al., 2017). Klasson et al. (2009) investigated the horizontal gene transfer between *Wolbachia* and the *Ae. aegypti* genome and concluded that the gene transfer most likely occurs from *Wolbachia* to the host genome. It is not possible to determine whether the *Wolbachia* genome sequence detected in this study originated from *Wolbachia*-infected mosquitoes or from the host-integrated *Wolbachia* genome. However, it is worth mentioning that this study identified contigs not only from a limited portion of the genomic regions of *Wolbachia* but also from numerous genome-wide regions (**Supplementary Table 4**). This finding supports the former possibility because, if the detected contigs were sequences of *Wolbachia* integrated into the mosquito genome, we would expect to observe fewer number of contigs from a narrower range of genomic regions that are integrated into the mosquito genome. Furthermore, ddRAD-Seq cannot completely eliminate the possibility of *Wolbachia* contamination from the environment or from the commensal or parasitic species such as nematodes within the mosquito’s body. This method detects *Wolbachia* based on the presence of DNA fragment from *Wolbachia* that are sequenced alongside DNA fragments from the host. It is impossible to determine whether the detected DNA fragments are from an authentic *Wolbachia* infection, contamination from other host species at larval stage or derived from the parasites within the mosquito’s body.

Another limitation of ddRAD-Seq is the less quantitative nature of the data. Using ddRAD-Seq makes accurate estimation of the relative *Wolbachia* gene concentrations per individual or population theoretically difficult, while it is possible using qPCR. However, in this study, we observed an interesting phenomenon: the number of reads mapped to the *Wolbachia* genome positively correlated with the number of *Wolbachia*-infected individuals (**Figures 2A–C**) or the relative density of *Wolbachia* in the *Ae. aegypti* population (**Figure 2D**; **Supplementary Figures 3A, B**). This result suggests the possibility of *Wolbachia* semi-quantification using ddRAD-Seq. However, ddRAD-Seq is theoretically unlikely to reflect the amount of *Wolbachia* in the template DNA due to PCR bias that might occur during library preparation. Future studies should continue to explore the possibility of using ddRAD-Seq data for the quantification of *Wolbachia* or other host organism-infecting bacteria (e.g., mosquitoes). For example, in this study, we found a pattern indicating that 3 of the 14 populations exhibited notably high numbers of reads (> 10,000 reads) mapped to the *Wolbachia w*AlbB genome (**Table 2**). Therefore, ddRAD-Seq could be potentially used as a tool to explore which local *Ae. aegypti* populations of *Wolbachia* could be potentially infected with a high prevalence rate.

Using ddRAD-Seq reads mapped to *Wolbachia* enabled us to discover significant differences in the levels of genetic diversity or evolutionary rates among different regions across the *Wolbachia* genome. This is a new discovery that would have likely remained undetected using simple PCR amplification and sequencing of only a part of the genome. The 150 SNP loci were expected to have a relatively fast evolutionary rate in the genome. Six of the 37 regions in the genome exhibited higher SNP numbers and genetic diversity than the other 31 regions (**STable 2**), suggesting higher mutation rates of the 6 regions (*RCSc_1* (catalytic activity), *trxB* (catalytic activity), *gph* (phosphoglycolate phosphatase activity), hypothetical protein (function not determined), *IS982 family transposase ISWpi16* (function not determined), and *IS481 family transposase ISWpi2* (nucleic acid binding) genes). To capture a wide range of genetically diverse *Wolbachia* strains, it is recommended to analyze a large number of loci, including those with high evolutionary rates (Held and Leese, 2007).

*Wolbachia* strains detected in this study were as follows: *w*Pip*, w*Ri*, TRS of Brugia malayi*, and *w*Mel. The ddRAD-Seq reads mapped in multiple genes across the *Wolbachia* genome, which might contribute to more accurate *Wolbachia* strain classification. The high evolutionary gene markers are useful for classifying *Wolbachia* into strains with phylogenetically high resolutions, such as subpopulations within populations. Such phylogenetically finer classification could contribute to unraveling how the arbovirus-blocking effect of different strains (e.g., DENV) might function (Flores et al., 2020), forecast the potential competition among different strains (Liang et al., 2020), and guide mass release programs. We could identify only two SNPs from the ddRAD-Seq reads in the conserved region of *16S* rRNA with 3.18E^-05^ nucleotide diversity, which is known for its low mutation rate and might not be appropriate for phylogenetic analysis (Held and Leese, 2007; Rodrigues and Silva, 2016; ColwelL and Haig, 2019). Only one read hit the *wsp* gene region, and no SNP was detected in the *wsp* gene. The *wsp* gene appears to be a fast-evolving gene marker and an informative gene for discriminating *Wolbachia* strains. The limited number of SNPs found in the *16S* rRNA gene could be attributed to its highly conserved nature. On the other hand, the absence of SNPs detected in the *wsp* gene may be due to the small number of ddRAD reads mapped to this region, resulting in an insufficient number of sequences for detecting sequence variations. In order to obtain a sufficient number of reads for mapping to the high number of genes across the *Wolbachia* genome, an optimization in the ddRAD-Seq library preparation (e.g., restriction enzyme selection) should be performed both for the host and the *Wolbachia* genomes. An alternative to the utilization of *16S* rRNA gene marker for *Wolbachia* detection, a study of Sankar et al. (2021) detected low prevalence of natural *Wolbachia* from *Anopheles culicifacies* and *Anopheles stephensi* using a nested PCR method of *16S* rRNA. Regarding the evolutionary rates of the *16S* rRNA and *wsp* markers, the detection of *Wolbachia* using PCR assays showed different results between targets genes where *wsp* gene were detected from higher number of individuals (83 individuals) than *16S* rRNA (49 individuals) (**Table 3**). *16S* rRNA gene is known to evolve at a slow rate, while the *wsp* gene is known to evolve at a fast rate. The use of fast evolving gene as a marker increases the possibility of false negative detection due to primer incompatibility in PCR. The different evolutionary rates of the two markers may have caused the difference in detection rates. Other published studies also reported different percentage of positive results for each marker (e.g., Wong et al., 2020) while defining true positive as positive for both. In general, PCR results require the use of at least two markers for detection to avoid bias and increase reliability.

In Metropolitan Manila, male *Ae. aegypti* yielded more reads that could be mapped to the *Wolbachia* genome than females (**Table 2**). This result indicates higher *Wolbachia* prevalence rates in males. The different feeding behavior and dispersal capabilities of female and male mosquitoes might affect the occurrence of *Wolbachia*. Female mosquitoes are hematophagous insects and tend to be anthropophagic (feeding on human blood), which might influence the composition of their gut microbiome. Sarma et al. (2022) reported that human blood-fed female *Ae. aegypti* exhibited higher microbiome species diversity than *Ae. aegypti* not fed human blood. The increased species diversity in the microbiome might increase competition among microorganisms, making it more difficult for *Wolbachia* to persist or multiply within the mosquito body. The fact that male mosquitoes prefer to stay close to mating sites and disperse less than females could be another factor limiting bacterial diversity (Minard et al., 2013). A high *Wolbachia* prevalence rate in four species of male mosquitoes was also identified in the study of Yang et al. (2021) in China using PCR. This study showed higher infection rates of *Wolbachia* in males than in females in other mosquito species: *Ae. albopictus* (male = 98.8%; female = 96.5%), *Armigeres subalbatus* (male = 98.1%; female = 93.2%), *Culex pipiens* (male = 95.7%; female = 80.4%), and *Culex tritaeniorhynchus* (male = 100%; female = 5.6%). Kulkarni et al. (2019) reported that male *Ae. aegypti* exhibited a higher infection rate than female mosquitoes in Florida (female = 3.6%, male = 5.5%) but a lower infection rate than female mosquitoes in New Mexico (female = 58.8%, male = 54.9%).

## 5 Conclusions

In this study, we demonstrated that ddRAD-Seq could be applied efficiently for detecting, quantifying, and assessing the genetic diversity of *Wolbachia* strains in *Ae. aegypti* populations in Metropolitan Manila, the Philippines. As expected, the ddRAD-Seq reads revealed various *Wolbachia* genes across the genome. This result suggests that ddRAD-Seq might complement the conventional PCR assays that detect *Wolbachia* relying only on a few DNA markers and provide more reliable support for genome-wide *Wolbachia* detection. Moreover, we demonstrated that the number of ddRAD-Seq reads mapped to the *Wolbachia* genome in each population tended to be consistent with the conventional PCR- and qPCR-based *Wolbachia* detection results. These results suggest the significance of further validating the quantitative assessment of *Wolbachia* infection by ddRAD-Seq in future studies. The prevalence and genetic diversity of the *Wolbachia* strains infecting the mosquito populations, as revealed by ddRAD-Seq, might provide useful insights into the design of a mass release program of *Ae. aegypti* artificially infected with *Wolbachia* for mosquito-borne disease control.

## Data availability statement

The datasets presented in this study can be found in online repositories. The names of the repository/repositories and accession number(s) can be found below: https://www.ncbi.nlm.nih.gov/, PRJNA954465.

## Ethics statement

The manuscript presents research on animals that do not require ethical approval for their study.

## Author contributions

AM was involved in conceptualizing, designing and performing the experiments, analyzing the data, and writing and editing the original draft. KW contributed to the conceptualization of the research, reviewed and edited the draft, and supervised the work. JR performed the qPCR experiments for the individual-based, reviewed, and edited the draft. NK was involved in supervising the data analysis. All authors contributed to the article and approved the submitted version.
