# Supplementary material for: Genome-wide detection of Wolbachia in natural Aedes aegypti populations using ddRAD-Seq
Source: Front Cell Infect Microbiol. 2023 Dec 14;13:1252656. doi: 10.3389/fcimb.2023.1252656 (PMC10755911; doi:10.3389/fcimb.2023.1252656)
Supplement: Supplementary file 1 [file DataSheet_1.docx]

Supplementary Material

Genome-wide detection of *Wolbachia* in natural *Aedes aegypti* populations using ddRAD-Seq

**Atikah Fitria Muharromah, Jerica Isabel L. Reyes, Ngure Kagia, Kozo Watanabe^*^**

*** Correspondence:** Corresponding Author: watanabe.kozo.mj@ehime-u.ac.jp

# Supplementary Figures and Tables

## Supplementary Figures

**Supplementary Figure 1.**DNA fragments distribution based on *in silico* digestion using seven combinations of restriction enzymes: *DraI-NlaIII* (blue), *MluCI-NlaIII* (red), *DraI-MluCI (*dark blue), *SbfIHF-MspI* (green*), EcoRI-NlaIII*(gray), *SbfIHF-HaeIII* (black), *SspI-NlaIII*(yellow).

| a)  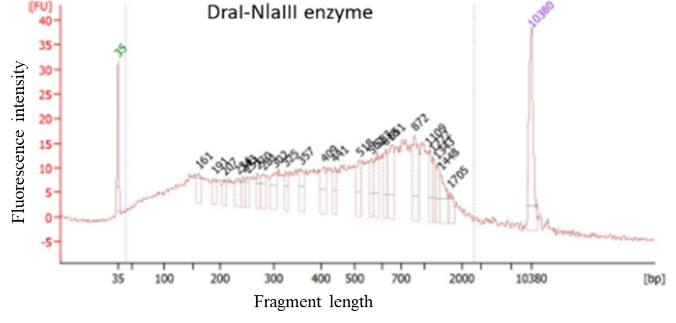 | b)  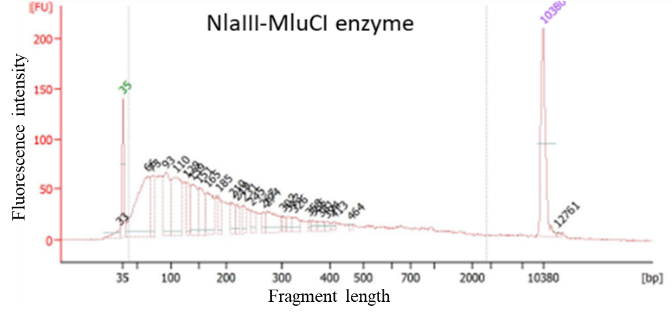 |
| --- | --- |
| c)  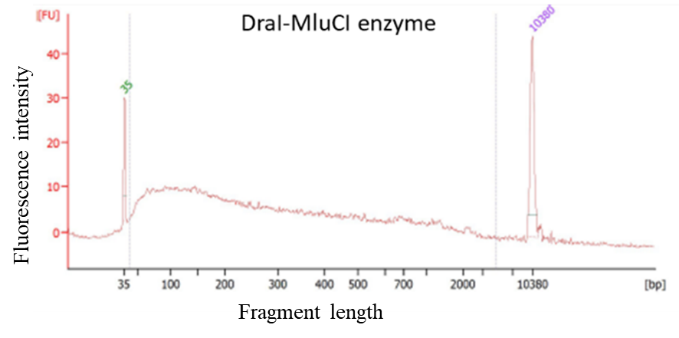 | d)  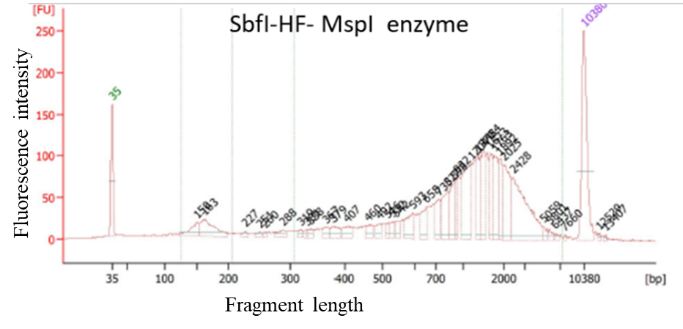 |
| e)  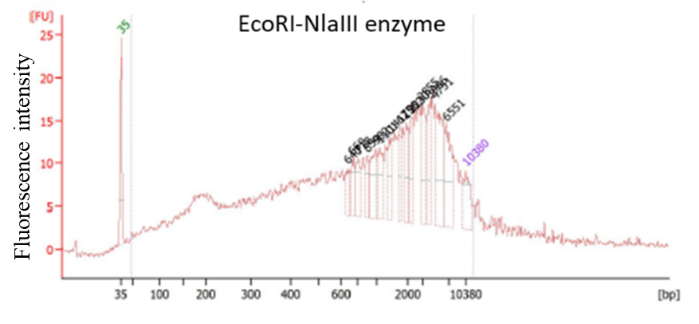 | f)  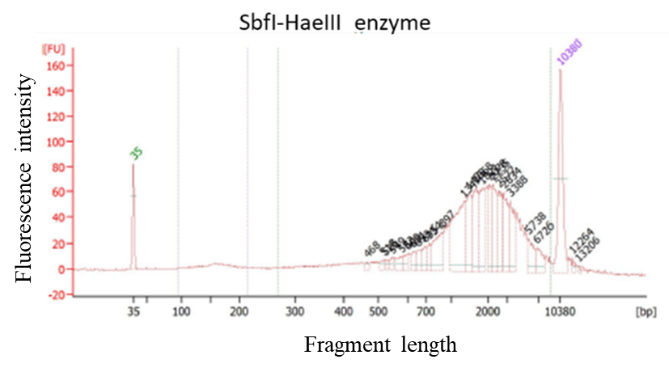 |
| g)  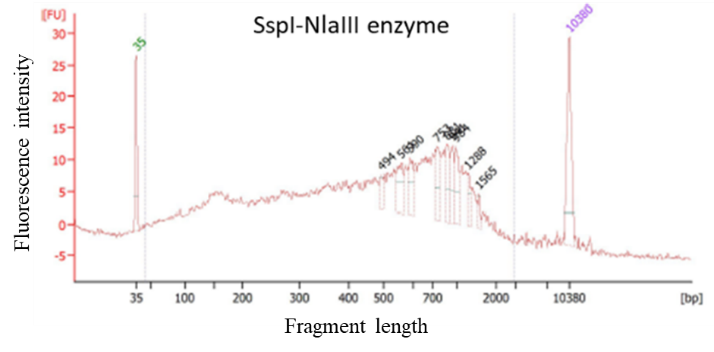 |  |

**Supplementary Figure 2.** DNA fragments distribution based on Bioanalyzer result (empirical digestion) using seven combination of restriction enzymes: *DraI-NlaIII* (a), *MluCI-NlaIII* (b), *DraI-MluCI* (c), *SbfIHF-MspI* (d), *EcoRI-NlaIII*(e), *SbfIHF-HaeIII* (f), *SspI-NlaIII*(g).

| 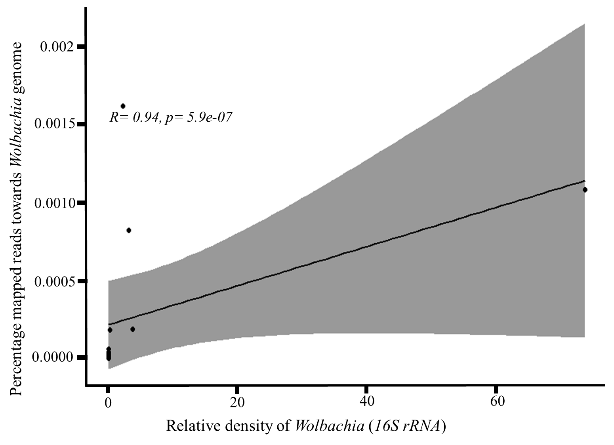  (a) | 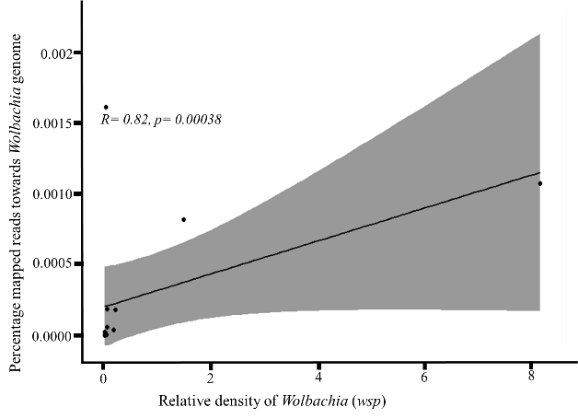  (b) |
| --- | --- |
| 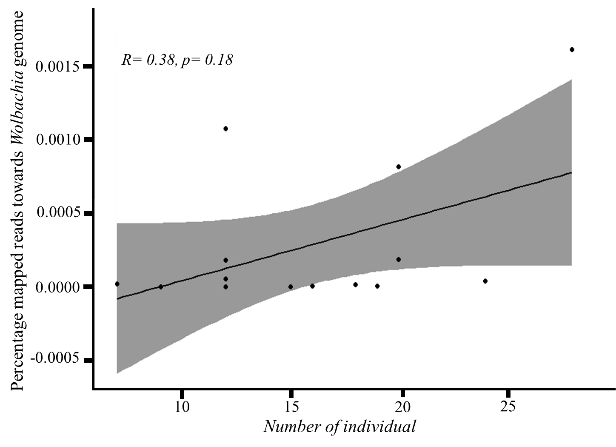  (c) | |
| **Supplementary Figure 3**. Correlation plot between reads that mapped to *Wolbachia* genome and the density of *Wolbachia* using *16S rRNA* gene (individual-based) (a), *wsp* gene (individual-based) (b) showed a significant correlation. The correlation plot between reads that mapped to *Wolbachia* genome and the total number of individuals per population showed no significant correlation. Supplementary tables **Supplementary** **Table 1**. *Wolbachia* spesies and strain identification result using MMSeqs2   \| **Population** \| **Total Reads** \| **Identification result** \| \| --- \| --- \| --- \| \| Female Central \| 6 \| unclassified *Wolbachia* \| \|  \| 10 \| *Wolbachia endosymbiont Culex quinquefasciatus Pel* \| \| Female North \| 2 \| identified until Genus level \| \|  \| 24 \| unclassified *Wolbachia* \| \|  \| 36 \| *Wolbachia endosymbiont Culex quinquefasciatus Pel* \| \|  \| 16 \| *Wolbachia endosymbiont of Drosophila melanogaster* \| \|  \| 4 \| *Wolbachia sp.* \| \|  \| 2 \| *Wolbachia sp. wRi* \| \|  \| 10 \| *Wolbachia pipientis* \| \| Female South \| 433 \| unclassified *Wolbachia* \| \|  \| 159 \| *Wolbachia endosymbiont Culex quinquefasciatus Pel* \| \|  \| 68 \| *Wolbachia endosymbiont of Drosophila melanogaster* \| \|  \| 34 \| *Wolbachia endosymbiont strain TRS of Brugia malayi* \| \|  \| 14 \| *Wolbachia sp. wRi* \| \|  \| 8 \| *Wolbachia sp.* \| \|  \| 18 \| *Wolbachia pipientis* \| \| Female West \| 8 \| *Wolbachia endosymbiont Culex quinquefasciatus Pel* \| \| Male Central \| 155 \| unclassified *Wolbachia* \| \|  \| 83 \| *Wolbachia endosymbiont Culex quinquefasciatus Pel* \| \|  \| 35 \| *Wolbachia endosymbiont of Drosophila melanogaster* \| \|  \| 10 \| *Wolbachia endosymbiont strain TRS of Brugia malayi* \| \|  \| 2 \| *Wolbachia pipientis* \| \| Male East \| 18 \| unclassified *Wolbachia* \| \|  \| 18 \| *Wolbachia endosymbiont Culex quinquefasciatus Pel* \| \|  \| 12 \| *Wolbachia endosymbiont of Drosophila melanogaster* \| \|  \| 3 \| *Wolbachia sp. wRi* \| \| Male North \| 2 \| unclassified *Wolbachia* \| \| Male South \| 6 \| identified until Genus level \| \|  \| 251 \| unclassified *Wolbachia* \| \|  \| 184 \| *Wolbachia endosymbiont Culex quinquefasciatus Pel* \| \|  \| 25 \| *Wolbachia sp. wRi* \| \|  \| 23 \| *Wolbachia endosymbiont strain TRS of Brugia malayi* \| \|  \| 13 \| *Wolbachia sp.* \| \|  \| 12 \| *Wolbachia pipientis* \| \| Male West \| 205 \| unclassified *Wolbachia* \| \|  \| 206 \| *Wolbachia endosymbiont Culex quinquefasciatus Pel* \| \|  \| 180 \| *Wolbachia endosymbiont of Drosophila melanogaster* \| \|  \| 35 \| *Wolbachia endosymbiont strain TRS of Brugia malayi* \| \|  \| 27 \| *Wolbachia sp. wRi* \| \|  \| 23 \| *Wolbachia sp.* \| \|  \| 22 \| *Wolbachia pipientis* \| | |

**Supplementary** **Table 2**. Genetic diversity and gene annotation in each SNP position in the genome with 1000 bp sliding window

| **Pos start** | **Pos end** | **Total SNP** | **π** | **Genes** |
| --- | --- | --- | --- | --- |
| 39,001 | 40,000 | 1 | 1.32E^-05^ |  |
| 67,001 | 68,000 | 2 | 6.36E^-05^ |  |
| 98,001 | 99,000 | 11 | 0.000227 | *RCSc_1* |
| 194,001 | 195,000 | 1 | 1.32E^-05^ |  |
| 411,001 | 412,000 | 2 | 2.65E^-05^ |  |
| 419,001 | 420,000 | 1 | 1.85E^-05^ |  |
| 457,001 | 458,000 | 3 | 3.98E^-05^ |  |
| 462,001 | 463,000 | 5 | 5.05E^-05^ |  |
| 535,001 | 536,000 | 6 | 6.39E^-05^ |  |
| 551,001 | 552,000 | 5 | 6.65E^-05^ |  |
| 599,001 | 600,000 | 1 | 2.12E^-05^ |  |
| 634,001 | 635,000 | 11 | 8.83E^-05^ | *trxB* |
| 659,001 | 660,000 | 4 | 3.19E^-05^ |  |
| 711,001 | 712,000 | 1 | 2.12E^-05^ |  |
| 749,001 | 750,000 | 1 | 1.32E^-05^ |  |
| 750,001 | 751,000 | 1 | 1.32E^-05^ |  |
| 762,001 | 763,000 | 1 | 1.32E^-05^ |  |
| 881,001 | 882,000 | 10 | 9.62E^-05^ | *gph* |
| 901,001 | 902,000 | 1 | 3.18E^-05^ |  |
| 909,001 | 910,000 | 1 | 2.65E^-06^ |  |
| 956,001 | 957,000 | 7 | 6.13E^-05^ |  |
| 962,001 | 963,000 | 2 | 2.65E^-05^ |  |
| 975,001 | 976,000 | 1 | 1.32E^-05^ |  |
| 1,027,001 | 1,028,000 | 2 | 2.12E^-05^ |  |
| 1,064,001 | 1,065,000 | 2 | 4.24E^-05^ |  |
| 1,121,001 | 1,122,000 | 5 | 0.000106 |  |
| 1,168,001 | 1,169,000 | 1 | 7.94E^-06^ |  |
| 1,173,001 | 1,174,000 | 2 | 3.18E^-05^ | *16s rRNA* |
| 1,210,001 | 1,211,000 | 1 | 1.32E^-05^ |  |
| 1,211,001 | 1,212,000 | 11 | 0.000184 | hypothetical protein |
| 1,260,001 | 1,261,000 | 4 | 5.31E^-05^ |  |
| 1,292,001 | 1,293,000 | 1 | 2.12E^-05^ |  |
| 1,331,001 | 1,332,000 | 1 | 1.32E^-05^ |  |
| 1,370,001 | 1,371,000 | 15 | 0.00033 | *IS982 family transposase ISWpi16* |
| 1,392,001 | 1,393,000 | 7 | 0.000104 |  |
| 1,431,001 | 1,432,000 | 18 | 0.000261 | *IS481 family transposase ISWpi2* |
| 1,447,001 | 1,448,000 | 1 | 2.38E^-05^ |  |

**Supplementary Table 3.** Bacteria diversity per population in the ddRAD-Seq data of *Ae.aegypti* mosquitoes from Metropolitan Manila with ≥ 0.0005 percentage of occurence.

| **Population** | **Bacteria diversity in Order level** |
| --- | --- |
| F central | Flavobacteriales, Enterobacterales, Lactobacillales, Hyphomicrobiales, Burkholderiales, Aquificales, Moraxellales, Vibrionales, Oceanospirillales, Bacillales, Pseudomonadales, Tissierellales, Rhodospirillales, Propionibacteriales, Chlamydiales, Rhodobacterales, Cytophagales, Neisseriales, Rickettsiales, Mycobacteriales, Sphingobacteriales, Xanthomonadales, Streptosporangiales, Chromatiales, Alteromonadales, Kitasatosporales, Bacteroidales, Eubacteriales, Rhodocyclales, Myxococcales, Sphingomonadales, Micrococcales, Spirochaetales, Legionellales, Cellvibrionales, Chitinophagales, Pseudonocardiales |
| F east | Hyphomicrobiales, Burkholderiales, Cytophagales, Flavobacteriales, Enterobacterales, Aeromonadales, Lactobacillales, Chitinophagales, Rhodospirillales, Bacillales, Pseudomonadales, Nevskiales, Neisseriales, Sphingomonadales, Moraxellales, Verrucomicrobiales, Mycobacteriales, Rhodobacterales, Nitrosomonadales, Sphingobacteriales, Alteromonadales, Vibrionales, Propionibacteriales, Rickettsiales, Rhodocyclales, Bdellovibrionales, Chromatiales, Kitasatosporales, Bacteriovoracales, Streptosporangiales, Legionellales, Xanthomonadales, Micrococcales, Oceanospirillales, Bacteroidales, Chlamydiales |
| F North | Hyphomicrobiales, Cytophagales, Burkholderiales, Enterobacterales, Rhodospirillales, Chitinophagales, Pseudomonadales, Neisseriales, Aeromonadales, Flavobacteriales, Rhodocyclales, Sphingomonadales, Bacteriovoracales, Rickettsiales, Lactobacillales, Sphingobacteriales, Oceanospirillales, Bacillales, Moraxellales, Nevskiales, Rhodobacterales, Nitrosomonadales, Verrucomicrobiales, Bdellovibrionales, Xanthomonadales, Micrococcales, Mycobacteriales, Alteromonadales, Frankiales, Kitasatosporales, Vibrionales, Eubacteriales, Propionibacteriales, Orbales, Caulobacterales, Aquificales, Legionellales, Chlamydiales, Desulfovibrionales, Chromatiales, Spirochaetales, Bacteroidales, Tissierellales, Opitutales, Phycisphaerales, Cellvibrionales, Streptosporangiales, Campylobacterales, Pseudonocardiales |
| F South | Hyphomicrobiales, Burkholderiales, Enterobacterales, Rickettsiales, Flavobacteriales, Bacillales, Lactobacillales, Nitrosomonadales, Moraxellales, Vibrionales, Propionibacteriales, Rhodospirillales, Rhodobacterales, Mycobacteriales, Pseudomonadales, Oceanospirillales, Frankiales, Alteromonadales, Chlamydiales, Xanthomonadales, Nevskiales, Cytophagales, Spirochaetales, Kitasatosporales, Eubacteriales, Aquificales, Streptosporangiales, Bacteroidales, Sphingomonadales, Rhodocyclales, Tissierellales, Micrococcales, Chitinophagales, Sphingobacteriales, Neisseriales, Phycisphaerales, Campylobacterales |
| F West | Hyphomicrobiales, Neisseriales, Burkholderiales, Flavobacteriales, Enterobacterales, Cytophagales, Pseudomonadales, Nitrosomonadales, Rhodospirillales, Moraxellales, Lactobacillales, Bacillales, Aeromonadales, Chlamydiales, Nevskiales, Sphingomonadales, Chitinophagales, Rhodobacterales, Propionibacteriales, Kitasatosporales, Vibrionales, Alteromonadales, Chromatiales, Rhodocyclales, Rickettsiales, Mycobacteriales, Bacteriovoracales, Sphingobacteriales, Verrucomicrobiales, Xanthomonadales, Micrococcales, Bacteroidales, Oceanospirillales, Tissierellales, Bdellovibrionales, Phycisphaerales |
| F North Manila | Enterobacterales, Flavobacteriales, Lactobacillales, Bacillales, Propionibacteriales, Burkholderiales, Moraxellales, Hyphomicrobiales, Pseudomonadales, Mycobacteriales, Rhodospirillales, Rhodobacterales, Vibrionales, Rickettsiales, Alteromonadales, Micrococcales, Cytophagales, Aquificales, Tissierellales, Chlamydiales, Streptosporangiales, Bacteroidales, Kitasatosporales, Xanthomonadales, Chitinophagales, Sphingomonadales, Phycisphaerales |
| F South Manila | Enterobacterales, Bacillales, Lactobacillales, Chlamydiales, Rhodobacterales, Moraxellales, Hyphomicrobiales, Mycobacteriales, Burkholderiales, Eubacteriales, Vibrionales, Propionibacteriales, Alteromonadales, Rhodospirillales, Pseudomonadales, Rickettsiales, Xanthomonadales, Flavobacteriales, Cytophagales, Bacteroidales, Micrococcales, Kitasatosporales, Sphingomonadales, Phycisphaerales, Chitinophagales, Aquificales, Spirochaetales, Pseudonocardiales |
| M Central | Hyphomicrobiales, Neisseriales, Burkholderiales, Flavobacteriales, Enterobacterales, Cytophagales, Pseudomonadales, Nitrosomonadales, Rhodospirillales, Moraxellales, Lactobacillales, Bacillales, Aeromonadales, Chlamydiales, Nevskiales, Sphingomonadales, Chitinophagales, Rhodobacterales, Propionibacteriales, Kitasatosporales, Vibrionales, Alteromonadales, Chromatiales, Rhodocyclales, Rickettsiales, Mycobacteriales, Bacteriovoracales, Sphingobacteriales, Verrucomicrobiales, Xanthomonadales, Micrococcales, Bacteroidales, Oceanospirillales, Tissierellales, Bdellovibrionales, Phycisphaerales |
| M East | Hyphomicrobiales, Burkholderiales, Flavobacteriales, Enterobacterales, Lactobacillales, Nitrosomonadales, Oceanospirillales, Neisseriales, Bacillales, Rickettsiales, Vibrionales, Cytophagales, Nevskiales, Moraxellales, Pseudomonadales, Chlamydiales, Rhodobacterales, Rhodospirillales, Alteromonadales, Aquificales, Propionibacteriales, Mycobacteriales, Sphingomonadales, Verrucomicrobiales, Kitasatosporales, Sphingobacteriales, Micrococcales, Xanthomonadales, Chitinophagales, Caulobacterales, Rhodocyclales, Aeromonadales, Chromatiales, Spirochaetales, Phycisphaerales, Tissierellales |
| M North | Hyphomicrobiales, Burkholderiales, Nitrosomonadales, Bacillales, Mycobacteriales, Lactobacillales, Enterobacterales, Aeromonadales, Chlamydiales, Nevskiales, Flavobacteriales, Moraxellales, Chromatiales, Cytophagales, Rhodospirillales, Xanthomonadales, Caulobacterales, Pseudomonadales, Rhodobacterales, Sphingomonadales, Rickettsiales, Bdellovibrionales, Neisseriales, Vibrionales, Propionibacteriales, Alteromonadales, Rhodocyclales, Chitinophagales, Cellvibrionales, Tissierellales, Kitasatosporales, Micrococcales, Sphingobacteriales, Phycisphaerales, Verrucomicrobiales, Oceanospirillales, Eubacteriales, Bacteriovoracales, Campylobacterales, Chroococcales, Aquificales |
| M South | Hyphomicrobiales, Burkholderiales, Rickettsiales, Nitrosomonadales, Lactobacillales, Enterobacterales, Bacillales, Nevskiales, Flavobacteriales, Moraxellales, Rhodospirillales, Rhodobacterales, Propionibacteriales, Xanthomonadales, Vibrionales, Cytophagales, Pseudomonadales, Mycobacteriales, Sphingomonadales, Kitasatosporales, Oceanospirillales, Sphingobacteriales, Cellvibrionales, Chitinophagales, Alteromonadales, Aquificales, Micrococcales, Tissierellales, Rhodocyclales, Neisseriales, Chromatiales, Bacteroidales, Phycisphaerales, Orbales |
| M West | Hyphomicrobiales, Burkholderiales, Rickettsiales, Lactobacillales, Bacillales, Enterobacterales, Chlamydiales, Rhodobacterales, Moraxellales, Mycobacteriales, Propionibacteriales, Vibrionales, Alteromonadales, Aquificales, Rhodospirillales, Pseudomonadales, Flavobacteriales, Cytophagales, Micrococcales, Xanthomonadales, Sphingomonadales, Tissierellales, Kitasatosporales, Oceanospirillales, Sphingobacteriales, Nitrosomonadales  Phycisphaerales |
| M North Manila | Chlamydiales, Lactobacillales, Bacillales, Enterobacterales, Hyphomicrobiales, Propionibacteriales, Burkholderiales, Rhodobacterales, Rhodospirillales, Mycobacteriales, Moraxellales, Aeromonadales, Alteromonadales, Oceanospirillales, Vibrionales, Pseudomonadales, Flavobacteriales, Rickettsiales, Sphingobacteriales, Sphingomonadales, Bacteroidales, Micrococcales, Xanthomonadales, Cytophagales, Rhodocyclales, Chitinophagales, Phycisphaerales, Tissierellales, Kitasatosporales, Eubacteriales, Aquificales, Campylobacterales |
| M South Manila | Enterobacterales, Moraxellales, Lactobacillales, Chlamydiales, Pseudomonadales, Bacillales, Burkholderiales, Propionibacteriales, Rhodobacterales, Mycobacteriales, Hyphomicrobiales, Alteromonadales, Vibrionales, Rickettsiales, Flavobacteriales, Rhodospirillales, Sphingobacteriales, Micrococcales, Xanthomonadales, Cytophagales, Chitinophagales, Kitasatosporales, Tissierellales, Aquificales, Eubacteriales, Phycisphaerales |

**Supplementary Table 4.** Number of *Wolbachia* contigs per *Ae.aegypti* population.

| Population | Number of *Wolbachia* contigs |
| --- | --- |
| F_Central | 35 |
| F_East | 3 |
| F_North | 89 |
| F_South | 296 |
| F_West | 10 |
| F_North_Manila | - |
| F_South Manila | 7 |
| M_Central | 193 |
| M_East | - |
| M_North | 6 |
| M_South | 423 |
| M_West | 504 |
| M_North_Manila | 5 |
| M_South_Manila | 6 |
